# Construction and validation of a multimodal MRI-based quantitative feature prediction model for the prognosis of non-metastatic primary osteosarcoma

**DOI:** 10.3389/fonc.2026.1787445

**Published:** 2026-04-22

**Authors:** Chao Xu, Chengcun Huo, Longjiang Wang

**Affiliations:** 1 Traumatic Orthopedics Department, East Campus of Zibo Central Hospital, Shandong, Zibo, China; 2 Imaging Department, Jinan Third People’s Hospital, Jinan, Shandong, China; 3 Imaging Department, Yantaishan Hospital, Yantai, Shandong, China

**Keywords:** diffusion-weighted imaging, dynamic contrast-enhanced MRI, multimodal MRI, non-metastatic primary osteosarcoma, prognostic prediction model, quantitative imaging features

## Abstract

**Purpose:**

Accurate prognosis assessment of non-metastatic primary osteosarcoma is essential for treatment decisions. This study aimed to develop and validate a pre-treatment predictive model using multimodal magnetic resonance imaging (MRI) quantitative parameters.

**Methods:**

This retrospective study included patients with non-metastatic primary osteosarcoma who received treatment at our hospital. Patients were divided into good or poor prognosis groups based on Response Evaluation Criteria in Solid Tumors at 30-month follow-up. We analyzed multimodal MRI data of these patients. Evaluated parameters included intramedullary extension measured by T2-weighted imaging, pure diffusion coefficient (D value), pseudo-diffusion coefficient (D* value), and apparent diffusion coefficient (ADC value) from diffusion-weighted imaging, and the contrast agent back-flux rate constant (Kep) from dynamic contrast-enhanced MRI. All these parameters were assessed as pre-treatment.

**Results:**

The training cohort included 169 good prognosis and 52 poor prognosis patients. Good prognosis patients showed significantly higher Kep (1.32 ± 0.24 vs 1.21 ± 0.21, P = 0.006), D value (0.95 ± 0.13 vs 0.84 ± 0.25, P = 0.003), D* value (19.78 ± 5.45 vs 17.34 ± 4.34, P = 0.004), and ADC value (1.11 ± 0.17 vs 1.01 ± 0.16, P<0.001), but lower intramedullary extension (9.62 ± 1.22 vs 10.50 ± 2.33, P = 0.012) compared to those with poor prognosis. The area under the curve (AUC) of the multivariate model integrating these features was 0.836. External validation confirmed the model’s discriminatory ability (AUC = 0.812) and reproduced significant differences in Kep, intramedullary extension, D value, D* value, and ADC value between groups.

**Conclusion:**

This study developed a predictive model based on multimodal MRI quantitative features that effectively identified poor prognosis in non-metastatic primary osteosarcoma, providing a non-invasive assessment tool to optimize treatment strategies.

## 1 Introduction

Osteosarcoma is the most common primary malignant bone tumor in adolescents and young adults, characterized by malignant mesenchymal cells that produce immature osteoid tissue (1, 2). Patients typically present with local pain, swelling, and sometimes pathological fractures (3). Treatment strategies for osteosarcoma generally include neoadjuvant chemotherapy, surgical resection, and adjuvant chemotherapy (4). However, outcomes for patients with non-metastatic disease can vary significantly, with a substantial portion facing poor prognosis due to recurrence or metastasis, largely influenced by tumor biology and varied treatment responses (5).

Traditional prognostic assessments have relied heavily on clinical staging systems such as the Enneking classification and postoperative histopathological evaluations of chemotherapy response. Unfortunately, these methods often fall short of providing timely predictive information, which is critical for personalized treatment planning (6). To enhance clinical decision-making, there remains a pressing need for more effective prognostic tools that can accurately predict patient outcomes prior to treatment initiation.

Emerging research has highlighted the importance of tumor microenvironment characteristics, including cellular composition, vascular architecture, and invasive potential, in determining the aggressiveness of osteosarcoma and its response to treatment (7, 8). The conventional Enneking staging system primarily focuses on histopathological features and anatomical location, thus neglecting the biological aggressiveness of tumors that advanced imaging techniques can capture (9). Recent studies have shown that diffusion-weighted imaging (DWI) and its derived parameters can provide quantitative insights into tissue cellularity and microcirculation perfusion (10). For example, apparent diffusion coefficient (ADC) and diffusion coefficient (D) values often correlate with cell density and tumor grade. Additionally, dynamic contrast-enhanced MRI (DCE-MRI) offers valuable insights into tumor biology, with parameters such as Ktrans (contrast agent transfer rate), Ve (extravascular extracellular volume fraction), and Kep (backflux rate constant) reflecting vascular permeability and endothelial integrity (11). Information extracted from anatomical sequences, such as T2-weighted imaging including intramedullary extension, further aids in quantifying tumor invasion within the bone marrow cavity (12). These MRI-derived features serve as non-invasive biomarkers that may reveal underlying biological behaviors associated with patient prognosis.

This study aims to develop and externally validate a predictive model based on multimodal MRI quantitative features to enhance prognostic accuracy for non-metastatic primary osteosarcoma. The primary objective is to identify key MRI features that can distinguish between patients with good and poor prognoses, construct a multivariate logistic regression prediction model incorporating these features, and rigorously evaluate the model’s performance and clinical utility both internally and externally. A unique aspect of this research involves integrating quantitative parameters from multiple advanced MRI sequences into a unified prognostic model, validated using an independent cohort. This approach aims to provide an objective pre-treatment assessment tool that facilitates early identification of high-risk patients, ultimately aiding in the formulation of personalized treatment intensification strategies or participation in new therapeutic trials, thereby improving patient survival rates.

## 2 Materials and methods

### 2.1 Case selection

This retrospective case-control study included 313 patients diagnosed with non-metastatic primary osteosarcoma at the Yantaishan Hospital from June 2021 to June 2024. Demographic information, clinical data, and multi-modal magnetic resonance imaging (MRI) quantitative parameters of the patients were collected through the case system. This study received approval from the ethics committee of the Yantaishan Hospital (No. LL-2025-207-L), and all procedures were conducted in accordance with the ethical standards outlined in the Helsinki Declaration of 1964 and its subsequent amendments. Since the study used fully anonymized patient data and posed no potential harm or impact on patient care, the ethics committee waived the requirement for informed consent from the patients.

### 2.2 Inclusion and exclusion criteria

The inclusion criteria are as follows: (1) Age range of 13–30 years old (The incidence of osteosarcoma in adolescents and young adults exhibits a bimodal distribution, with the first peak occurring between the ages of 13 and 30. Patients within this age range represent the core population of non-metastatic osteosarcoma. The tumor biology, treatment protocols, and clinical outcome patterns in this age group are relatively homogeneous, which helps to minimize confounding factors due to extreme age differences and allows the study to focus more effectively on assessing the independent prognostic value of quantitative imaging features). (2) Patients were initially diagnosed with non-metastatic primary osteosarcoma at our hospital. The diagnosis was established in accordance with contemporary diagnostic criteria, which require a combination of characteristic radiographic/MRI findings and definitive histopathological evidence from biopsy (13). Metastatic disease was ruled out by standard staging investigations (bone scan and chest CT) at initial presentation. (3) Patients who underwent extensive tumor resection surgery (14). (4) Complete multi-modal MRI radiological examination data. (5) Complete medical records and follow-up records.

The exclusion criteria are as follows: (1) Patients who only received palliative surgery or had distant metastasis. (2) Neurological diseases or cognitive impairments. (3) Previous history of treatment for malignant tumors. (4) Contraindications to contrast agents. (5) Pregnant or lactating women. (6) Incomplete or missing critical data necessary for the study.

### 2.3 Grouping criteria

After the completion of patient treatment, the prognosis was assessed according to the Response Evaluation Criteria in Solid Tumors (RECIST) at the 2.5-year follow-up. RECIST is an objective criterion widely used in oncology clinical trials to quantify treatment response by measuring changes in the diameter of target lesions. In osteosarcoma, radiological responses defined by RECIST have been confirmed to correlate with pathological response rates and event-free survival (EFS), making it a reliable surrogate endpoint for assessing treatment response (15, 16). The selection of 30 months as the evaluation time point is based on the clinical course characteristics of osteosarcoma. According to the EURAMOS-1 trial and the ESMO Clinical Practice Guidelines, the majority of disease recurrences and metastatic events in high-grade osteosarcoma occur within the first 2–3 years after diagnosis (17, 18). Therefore, the treatment response status at 30 months serves as a clinically meaningful surrogate endpoint that is closely related to long-term survival outcomes in this patient population. Patients with progressive disease (PD) or death were included in the poor prognosis group (n=52), while those with complete response (CR), partial response (PR), and stable disease (SD) were placed in the good prognosis group (n=169). The treatment response was defined as: CR means patients with complete resolution of the target lesions; PR means patients with >30% decline of target tumor diameter; PD means patients with ≥20% elevation of target tumor diameter; SD means the target tumor’s diameter between PR and PD. Additionally, 92 patients who met the same inclusion criteria were included for external validation. During the 15-month follow-up period, these patients were also evaluated for their prognosis according to RECIST and were divided into a poor prognosis group (n=27) and a good prognosis group (n=65).

### 2.4 Imaging procedures

MRI data were acquired using a 3T whole-body scanner (Discovery 750w, GE Healthcare, USA) located in the hospital’s radiology department before treatment. To ensure the stability and reproducibility of imaging data, the scanner undergoes standard system calibration and quality control procedures each day before use. These procedures include routine shimming and the use of a standard water phantom to verify signal-to-noise ratio (SNR) and geometric distortion. All sequence parameters are within the acceptable range for quality control.

A standard head coil was used for both transmission and reception. All patients followed a standardized preparation protocol before scanning to ensure consistent conditions. Patients were required to fast for at least 4 hours prior to the scan but were allowed to drink water freely. Before the administration of gadolinium contrast agent, it was confirmed that they had no known allergies and that their renal function met safety standards. Technicians provided detailed explanations of the examination process and precautions to alleviate anxiety and ensure patient cooperation. They lay in a relaxed state without sleeping or engaging in cognitive activities. All subjects wore earplugs to reduce external auditory stimuli, and their heads were immobilized to minimize potential motion artifacts.

Patients received T1-weighted Imaging. The following parameters: Flip Angle 2° and 15°, repetition time (TR) 3 ms, echo time (TE) 1.3 ms, field of view (FOV)240 cm, layer thickness 3mm and layer spacing 0.5 mm. The matrix size was appropriately set to achieve isotropic or near-isotropic spatial resolution, in order to meet the requirements for precise anatomical localization and volumetric measurements. The data was analyzed using Cine tool package (GE Healthcare, USA). The parameters of contrast agent transfer rate between blood and tissue (K^trans^), contrast agent back-flux rate constant (K_ep_), extravascular extracellular fractional volume (V_e_) were recorded. The following parameters were used to acquire magnetization-prepared three-dimensional T2-weighted fast gradient echo sagittal images: TR: 3900 ms, TE: 82 ms, matrix: 256×256. This parameter setting aims to achieve good bone marrow-tumor contrast and clearly display the extent of intramedullary invasion. The diffusion-weighted imaging (DWI) scanning parameters are as follows: TR 6000 ms, TE 70 ms, FOV 400 mm × 300 mm, slice thickness of 5 mm, interslice gap of 1 mm. The diffusion sensitivity coefficient b-values were set to 0 and 1000 s/mm^2^, with diffusion-sensitive gradient fields applied along the X, Y, and Z axes. The parameters are based on their extensive validation in bone and soft tissue tumors. They are capable of suppressing the T2 shine-through effect while providing good sensitivity to changes in cell density, making them suitable for calculating the apparent diffusion coefficient (ADC). DWI were acquired with diffusion gradient encoding in 3 orthogonal directions with b values (0 and 700 s/mm^2^). In all images, a fat-saturated pulse was used to exclude chemical-shift artifacts.

### 2.5 Diameter measurement and volume calculation of tumor

**Figure 1:**
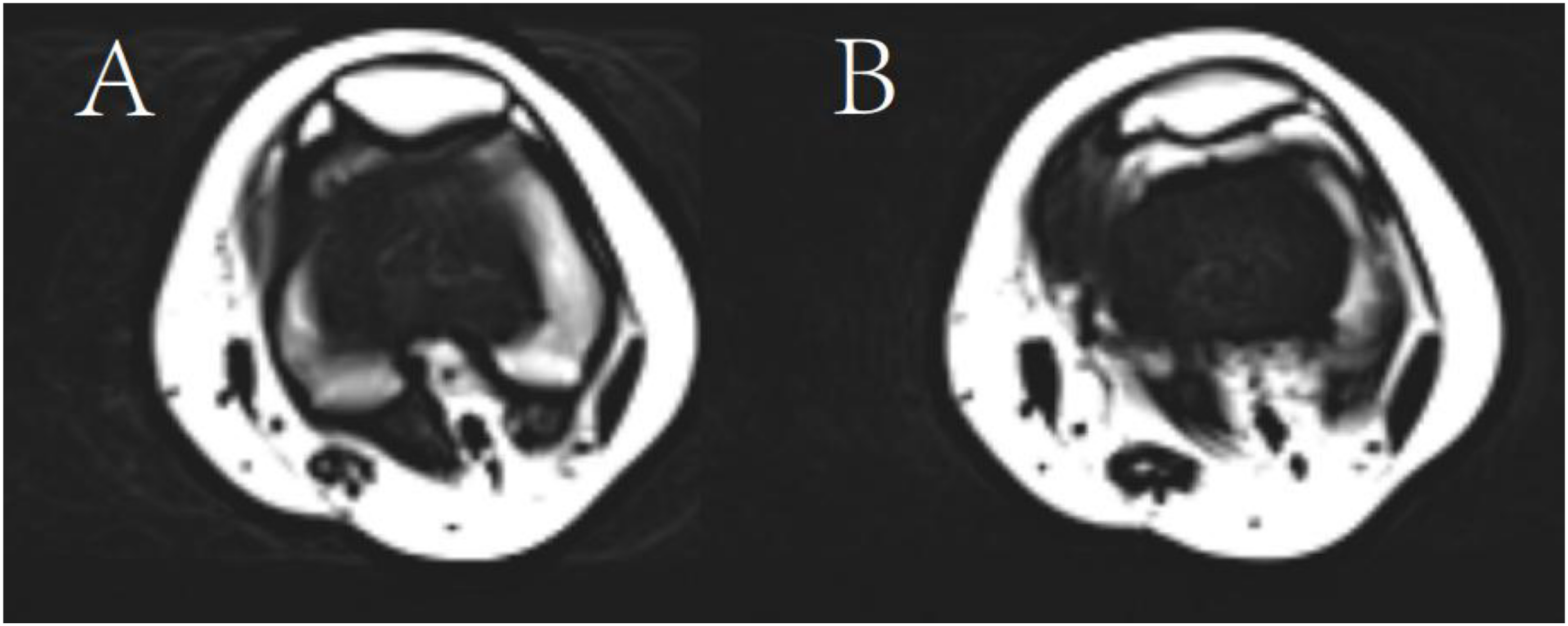
T2-weighted sequence. **(A)** Good Prognosis; **(B)** Poor Prognosis.

Three-dimensional tumor diameters of each patient were measured on T2-weighted images (**Figure 1**). Tumor volume (TV) was calculated from the largest tumor length (TL), tumor width (TW), and tumor depth (TD) (19). Tumor volumes of ellipsoidal tumors with soft tissue extension were calculated using the formula TV = 0.53×TL×TW×TD; tumor volumes of cylindric tumors without soft tissue extension were calculated using the formula TV = 0.785×TL×TW×TD (20). All imaging measurements were independently performed by a radiologist with over five years of experience in musculoskeletal imaging diagnosis. The radiologist was blinded to the patients’ clinical grouping.

### 2.6 Image postprocessing

**Figure 2:**
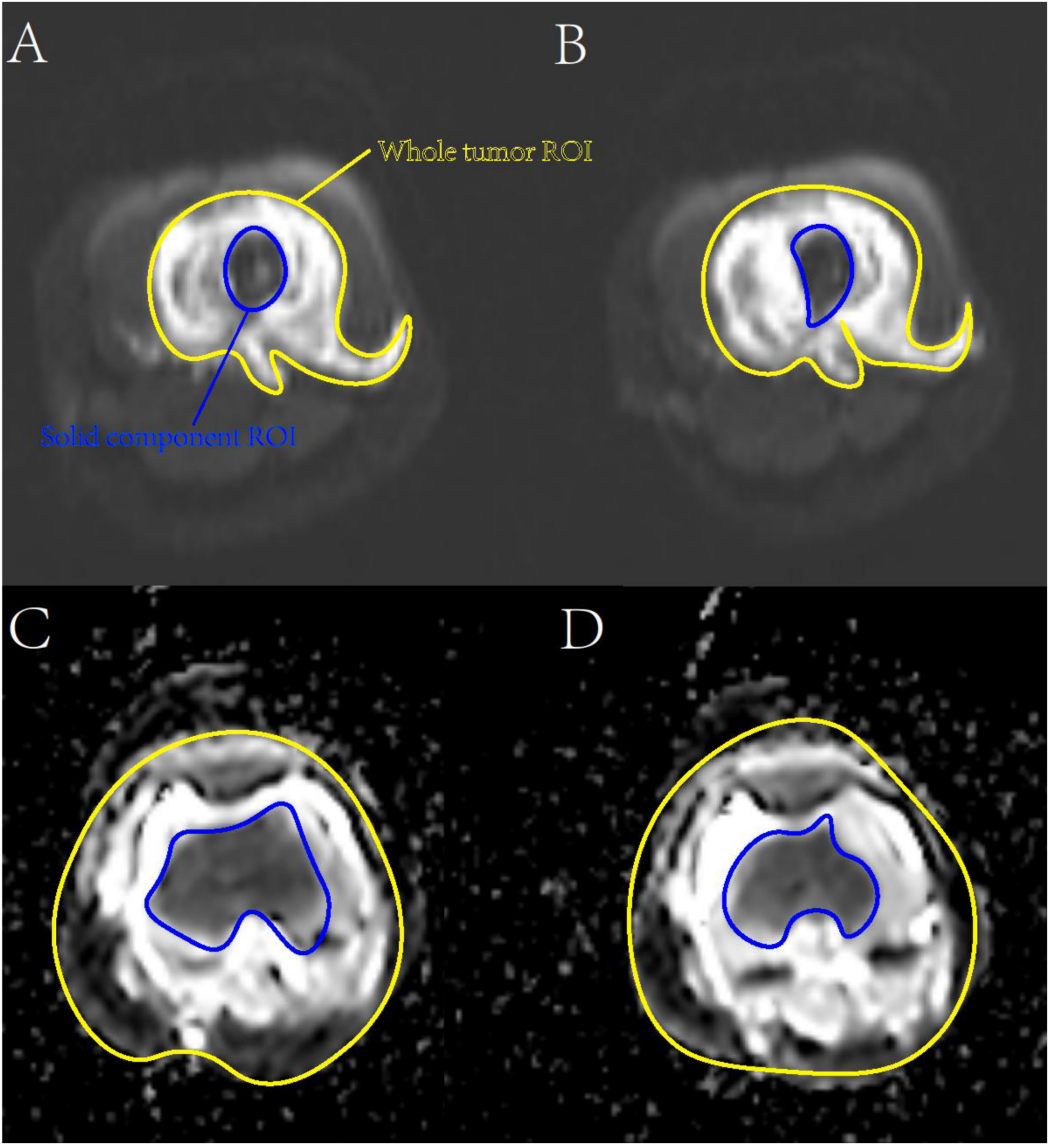
DWI map and ADC map. **(A)** DWI map of good prognosis; **(B)** DWI map of poor prognosis; **(C)** ADC map of good prognosis; **(D)** ADC map of poor prognosis.

The raw data were transferred to a computer workstation (Sun Microsystems, ADW4.2), where the DWI data was processed using Func tool 2 image analysis software (GE Medical Systems, USA) to obtain the pre- and post-treatment tumor ADC maps and ADC histograms (**Figure 2**). The acquisition of ADC values followed a standardized protocol. A radiologist with over five years of experience in musculoskeletal imaging diagnosis manually delineated regions of interest (ROIs) on a workstation under the guidance of multi-modal image fusion. To assess the reproducibility of ROI delineation, the same radiologist and another radiologist with three years of experience independently re-delineated ROIs for 30 randomly selected cases. The intra-class correlation coefficients (ICC) for each parameter were calculated. The intra-observer ICC for all parameters ranged from 0.821 to 0.934, and the inter-observer ICC ranged from 0.796 to 0.905, indicating good reproducibility. Solid region ROIs were primarily selected based on areas with obvious homogeneous enhancement on contrast-enhanced T1-weighted images, while also referring to T2-weighted images and high b-value (b=1000 s/mm²) DWI images to avoid areas of visually discernible necrosis, cystic changes, hemorrhage, and peritumoral edema. Necrotic region ROIs were delineated on non-enhancing areas in contrast-enhanced T1-weighted images that showed markedly high signal intensity on T2-weighted images. Whole-lesion ROIs were traced along the boundaries of the tumor parenchyma (including both solid and necrotic components) on consecutive axial T2-weighted or contrast-enhanced T1-weighted images, aiming to encompass the entire tumor volume. Subsequently, the software automatically mapped all these ROIs onto the corresponding ADC maps and calculated the mean ADC values for each region. The difference between the features extracted from pre-treatment tumor ADC histograms were also observed. The following parameters were recorded: pure diffusion coefficient (D value), pseudo-diffusion coefficient (D* value), ADC and the perfusion fraction (f value).

### 2.7 General information

The specific definitions of the Enneking staging are as follows: IA (low grade, intracompartmental) refers to a tumor that is low-grade malignant and is completely located within the anatomical compartment of the bone; IB (low grade, extracompartmental) indicates that although the tumor is low-grade malignant, it has invaded the surrounding soft tissues; IIA (high grade, intracompartmental) refers to a high-grade malignant tumor confined to the bone compartment; IIB (high grade, extracompartmental) represents a high-grade malignant tumor that extends into the surrounding soft tissues (21). Tumor sites were determined by radiologists using MRI images, which provided detailed anatomical information for accurate localization of the tumor. Common sites included femur, tibia/fibula, humerus, pelvis, head/neck, and other locations. Pathological types were classified into osteoblastic and others based on histopathological examination. Osteoblastic type refers to tumors that predominantly produce bone matrix, while ‘others’ include fibroblastic, chondroblastic, and telangiectatic types among others.

### 2.8 Statistical method

Data analysis was performed using SPSS 29.0 statistical software (SPSS Inc., Chicago, IL, USA). Categorical data were presented in the format [n (%)]. Chi-square tests were used when the sample size was ≥40 and the theoretical frequency (T) ≥5, with the test statistic expressed as chi-square. If the sample size was ≥40 but the theoretical frequency fell within the range of 1≤T<5, the correction formula was applied to adjust the chi-square test. For sample sizes less than 40 or when the theoretical frequency T was less than 1, Fisher’s exact probability method was employed for statistical analysis. Continuous data that followed a normal distribution were reported as (mean ± standard deviation) and compared using t-tests. Pearson correlation analysis was used for continuous variables, while Spearman correlation analysis was applied for categorical variables.

All multi-modal MRI quantitative parameters were first evaluated for their independent association with poor prognosis using univariate logistic regression analysis. Variables with P < 0.05 in the univariate analysis were included in the multivariate logistic regression model, with variable selection performed using a forward stepwise approach based on the likelihood ratio. The final model retained only statistically significant independent predictors.

The discrimination ability of the model was assessed using the area under the receiver operating characteristic curve (AUC), and calibration was evaluated using the Hosmer-Lemeshow goodness-of-fit test and Brier score. The results of the logistic regression analysis are presented as odds ratios (OR) with corresponding 95% confidence intervals (CI) and p-values. A p-value < 0.05 was considered statistically significant.

## 3 Results

### 3.1 Analysis of differences in general information of patients

When comparing the demographic characteristics between the Good Prognosis Group and Poor Prognosis Group, most parameters showed no significant differences, as indicated by several P values (**Table 1**). Age showed no significant difference (P = 0.955), nor did gender (P = 0.955), BMI (P = 0.163), family history (P = 0.985), residence (P = 0.792), Han ethnicity (P = 0.984), smoking status (P = 0.415), or drinking habits (P = 0.321). Education level approached significance but ultimately did not reach the threshold for statistical significance (P = 0.096). These results suggest that demographic characteristics are similarly distributed between patients with good and poor prognoses in this study cohort.

**Table 1:** Comparison of demographics characteristics between two groups of patients.

Parameters	Good prognosis group (n=169)	Poor prognosis group (n=52)	t/χ^2^	P
Age (years) [n (%)]			0.003	0.955
- <18	87 (51.48%)	27 (51.92%)		
- ≥18	82 (48.52%)	25 (48.08%)		
BMI (kg/m^2^)	22.45 ± 3.00	23.13 ± 3.30	1.398	0.163
Gender [n (%)]			0.003	0.955
- Male	69 (40.83%)	21 (40.38%)		
- Female	100 (59.17%)	31 (59.62%)		
Education level [n (%)]			2.778	0.096
- Secondary School or below	97 (57.40%)	23 (44.23%)		
- College or above	72 (42.60%)	29 (55.77%)		
Family History [n (%)]	12 (7.10%)	3 (5.77%)	0.001	0.985
Residence [n (%)]			0.070	0.792
- Urban	117 (69.23%)	37 (71.15%)		
- Rural	52 (30.77%)	15 (28.85%)		
Han ethnicity [n (%)]	157 (92.90%)	48 (92.31%)	0.002	0.984
Smoking [n (%)]	31 (18.34%)	7 (13.46%)	0.666	0.415
Drinking [n (%)]	33 (19.53%)	7 (13.46%)	0.987	0.321

BMI, Body Mass Index.

When comparing the clinical characteristics between the Good Prognosis Group and Poor Prognosis Group, most parameters showed no significant differences (**Table 2**). The site of the tumor did not differ significantly (P = 0.892), nor did Enneking stage (P = 0.691), the presence of a pathological fracture (P = 0.137), disease duration (P = 0.654), or follow-up period (P = 0.332). Although pathology approached a significant difference (P = 0.071), it did not reach the threshold for statistical significance. These findings indicate that clinical characteristics are largely similar between patients with good and poor prognoses in this study cohort, suggesting that factors other than those examined here may be more influential in determining prognosis.

**Table 2:** Comparison of clinical characteristics between two groups of patients.

Parameters	Good prognosis group (n=169)	Poor prognosis group (n=52)	t/χ^2^	P
Site of tumor [n (%)]			0.123	0.892
- Femur	89 (52.66%)	29 (55.77%)		
- Tibia/fibula	42 (24.85%)	14 (26.92%)		
- Humerus	19 (11.24%)	4 (7.69%)		
- Pelvis	11 (6.51%)	2 (3.85%)		
- Head/neck	6 (3.55%)	3 (5.77%)		
- Other	2 (1.18%)	0 (0.00%)		
Enneking stage [n (%)]			1.461	0.691
- IA	17 (10.06%)	8 (15.38%)		
- IB	36 (21.30%)	12 (23.08%)		
- IIA	65 (38.46%)5	19 (36.54%)		
- IIB	1 (30.18%)	13 (25.00%)		
Pathological fracture [n (%)]	35 (20.71%)	6 (11.54%)	2.214	0.137
Pathology [n (%)]			3.261	0.071
- Osteoblastic	43 (25.44%)	7 (13.46%)		
- Others	126 (74.56%)	45 (86.54%)		
Course of Disease (months)	4.46 ± 1.56	4.35 ± 1.57	0.449	0.654
Follow-up period (months)	18.24 ± 1.03	18.41 ± 1.15	0.972	0.332

### 3.2 Comparison of multimodal MRI quantitative features between two groups of patients

**Figure 3:**
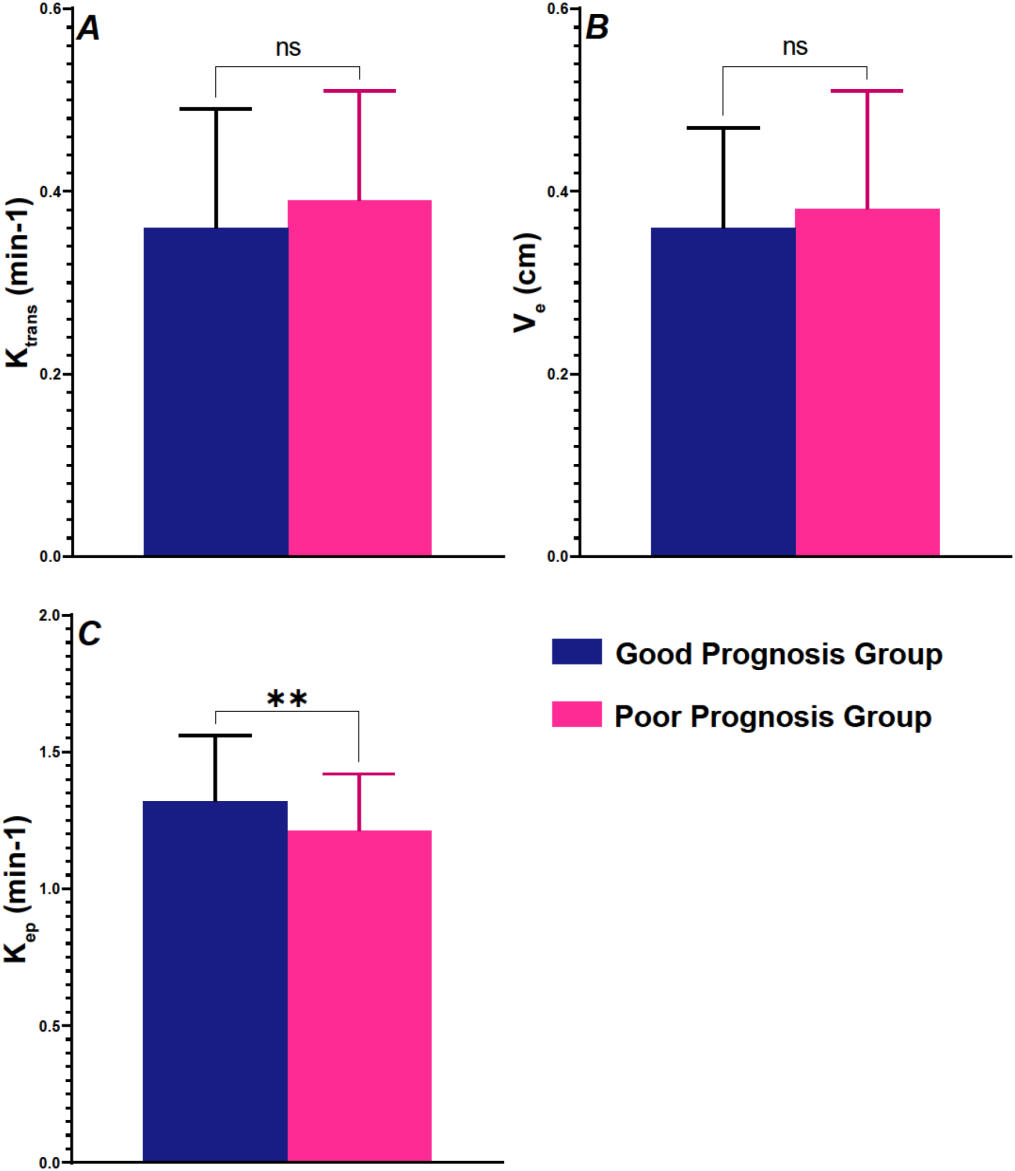
Comparison of T1 features between two groups of patients. **(A)** K^trans^ (min^-1^); **(B)** V_e_ (cm); **(C)** K_ep_ (min^-1^). T1, T1-Weighted Imaging; K^trans^, contrast agent transfer rate between blood and tissue; V_e_, extravascular extracellular fractional volume; K_ep_, contrast agent back-flux rate constant. ns, no significant; **P<0.01.

In the comparison of T1 features between the Good Prognosis Group and Poor Prognosis Group, significant differences were observed for the contrast agent back-flux rate constant (Kep; **Figure 3**). Kep was significantly higher in the Good Prognosis Group compared to the Poor Prognosis Group (P = 0.006). For the other parameters, no significant differences were found. The contrast agent transfer rate between blood and tissue (Ktrans) did not show a significant difference between the two groups (P = 0.128), nor did the extravascular extracellular fractional volume (Ve) (P = 0.539).

**Figure 4:**
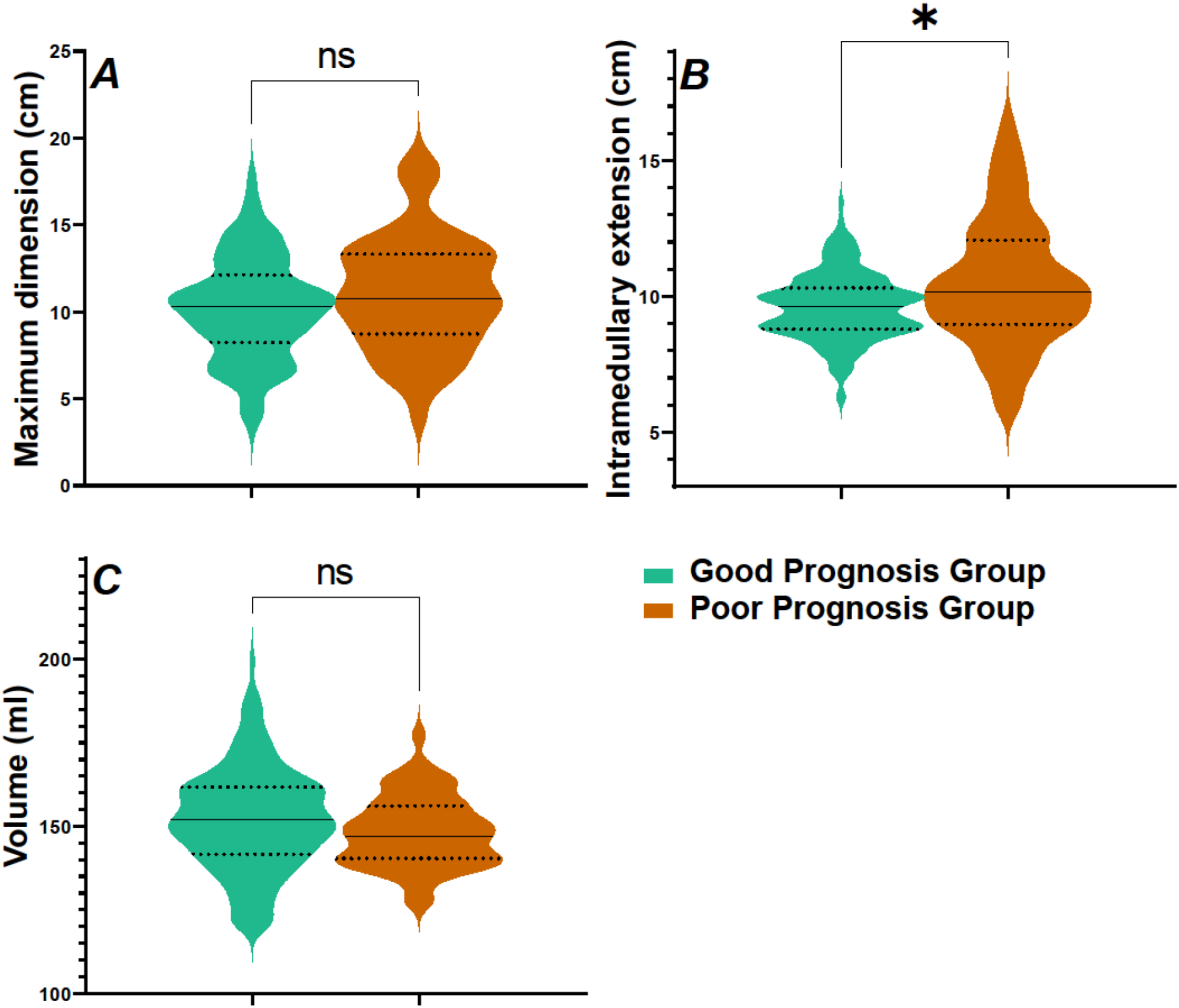
Comparison of T2 features between two groups of patients. **(A)** Maximum dimension (cm); **(B)** Intramedullary extension (cm); **(C)** Volume (ml); T2, T2-Weighted Imaging. ns, no significant; *P<0.05.

In the comparison of T2 features between the Good Prognosis Group and Poor Prognosis Group, significant differences were observed for intramedullary extension (**Figure 4**). Specifically, intramedullary extension was significantly greater in the Poor Prognosis Group compared to the Good Prognosis Group (P = 0.012). For the other parameters, no significant differences were found. Maximum dimension did not show a significant difference between the two groups (P = 0.078), nor did the volume (P = 0.059).

In the analysis of DWI features between the Good Prognosis Group and Poor Prognosis Group, significant differences were observed for several parameters (**Table 3**). The pure diffusion coefficient (D value) was significantly higher in the Good Prognosis Group compared to the Poor Prognosis Group (P = 0.003). The pseudo-diffusion coefficient (D* value) was also significantly higher in the Good Prognosis Group than in the Poor Prognosis Group (P = 0.004). Additionally, the Apparent Diffusion Coefficient (ADC value) was significantly higher in the Good Prognosis Group relative to the Poor Prognosis Group (P<0.001). However, the perfusion fraction (f value) did not show a significant difference between the two groups (P = 0.227). These results indicate that patients with a good prognosis exhibited significantly higher values for D value, D* value, and ADC value compared to those with poor prognosis.

**Table 3:** Analysis of DWI features between the two groups of patients.

Parameters	Good prognosis group (n=169)	Poor prognosis group (n=52)	t	P
D value (×10–^3^ mm^2^/s)	0.95 ± 0.13	0.84 ± 0.25	3.101	0.003
D* value (×10–^3^ mm^2^/s)	19.78 ± 5.45	17.34 ± 4.34	2.949	0.004
ADC value (×10–^3^ mm^2^/s)	1.11 ± 0.17	1.01 ± 0.16	3.610	<0.001
f value	0.31 ± 0.12	0.29 ± 0.11	1.211	0.227

DWI, Diffusion-Weighted Imaging; D value, pure diffusion coefficient; D* value, pseudo-diffusion coefficient; ADC, Apparent Diffusion Coefficient; f value, perfusion fraction.

### 3.3 Correlation analysis

In the correlation analysis between multimodal MRI quantitative features and poor prognosis of non-metastatic primary osteosarcoma, significant P values were observed for Kep (P = 0.006), intramedullary extension (P = 0.011), D value (P<0.001), D* value (P = 0.003), and ADC value (P<0.001; **Table 4**). These results indicate that these MRI quantitative features are significantly correlated with poor prognosis, suggesting their potential utility as prognostic markers in the clinical assessment of non-metastatic primary osteosarcoma.

**Table 4:** Correlation analysis between multimodal MRI quantitative features on poor prognosis of non-metastatic primary osteosarcoma.

Variable	rho	P
K_ep_	-0.183	0.006
Intramedullary extension	0.172	0.011
D value	-0.241	<0.001
D* value	-0.200	0.003
ADC value	-0.241	<0.001

MRI, Magnetic Resonance Imaging; K_ep_, contrast agent back-flux rate constant; D value, pure diffusion coefficient; D* value, pseudo-diffusion coefficient; ADC, Apparent Diffusion Coefficient.

### 3.4 Regression analysis of poor prognosis of non-metastatic primary osteosarcoma

In the univariate and multivariate regression analysis of multimodal MRI quantitative features on poor prognosis of non-metastatic primary osteosarcoma, significant P values were observed for all parameters in both analyses (**Table 5**). For univariate analysis: Kep (P = 0.007), intramedullary extension (P<0.001), D value (P<0.001), D* value (P = 0.004), and ADC value (P<0.001). In the multivariate analysis, similar significance was found: Kep (P = 0.003), intramedullary extension (P = 0.002), D value (P<0.001), D* value (P = 0.042), and ADC value (P = 0.006). These findings indicate that these MRI quantitative features are strongly associated with poor prognosis, even after adjusting for potential confounders, highlighting their potential as independent prognostic markers in non-metastatic primary osteosarcoma.

**Table 5:** **|**Univariate and multivariate regression analysis of multimodal MRI quantitative features on poor prognosis of non-metastatic primary osteosarcoma.

Parameters	Univariate analysis			Multivariate analysis		
	P	OR	95%CI	P	OR	95%CI
K_ep_	0.007	0.136	0.031- 0.555	0.003	0.073	0.013-0.404
Intramedullary extension	<0.001	1.401	1.153-1.725	0.002	1.416	1.138-1.763
D value	<0.001	0.023	0.003-0.149	<0.001	0.016	0.002-0.133
D* value	0.004	0.911	0.852-0.969	0.042	0.928	0.863-0.997
ADC value	<0.001	0.035	0.005-0.227	0.006	0.047	0.005-0.415

MRI, Magnetic Resonance Imaging; K_ep_, contrast agent back-flux rate constant; D value, pure diffusion coefficient; D* value, pseudo-diffusion coefficient; ADC, Apparent Diffusion Coefficient; OR, Odds Ratio; CI, Confidence Interval.

### 3.5 ROC curve analysis of multimodal MRI quantitative features on poor prognosis of non-metastatic primary osteosarcoma

In the ROC curve analysis of multimodal MRI quantitative features on poor prognosis of non-metastatic primary osteosarcoma, the AUC values indicated moderate discriminatory power for all parameters (**Table 6**). Specifically, Kep had an AUC of 0.625, intramedullary extension 0.617, D value 0.664, D* value 0.636, and ADC value 0.664. These results suggest that while each parameter shows some ability to distinguish between good and poor prognosis, none of them exhibit excellent discrimination. The Youden index ranged from 0.241 to 0.340, with D value showing the highest value (0.340), indicating it as the relatively best among the individual parameters in this context. Overall, these findings highlight the potential utility of these MRI quantitative features in predicting prognosis, though further refinement may be necessary to improve their diagnostic accuracy.

**Table 6:** ROC curve analysis of multimodal MRI quantitative features on poor prognosis of non-metastatic primary osteosarcoma.

Parameters	Best threshold	Sensitivities	Specificities	AUC	Youden index	F1 score
K_ep_	1.355	0.808	0.456	0.625	0.264	0.144
Intramedullary extension	11.795	0.288	0.953	0.617	0.241	0.400
D value	0.865	0.577	0.763	0.664	0.340	0.217
D* value	19.635	0.750	0.533	0.636	0.283	0.168
ADC value	1.065	0.673	0.621	0.664	0.294	0.195

MRI, Magnetic Resonance Imaging; K_ep_, contrast agent back-flux rate constant; D value, pure diffusion coefficient; D* value, pseudo-diffusion coefficient; ADC, Apparent Diffusion Coefficient; AUC, Area Under the Curve; ROC, Receiver Operating Characteristic.

### 3.6 Development of a prediction model for multimodal MRI quantitative features influencing poor prognosis of non-metastatic primary osteosarcoma

**Figure 5:**
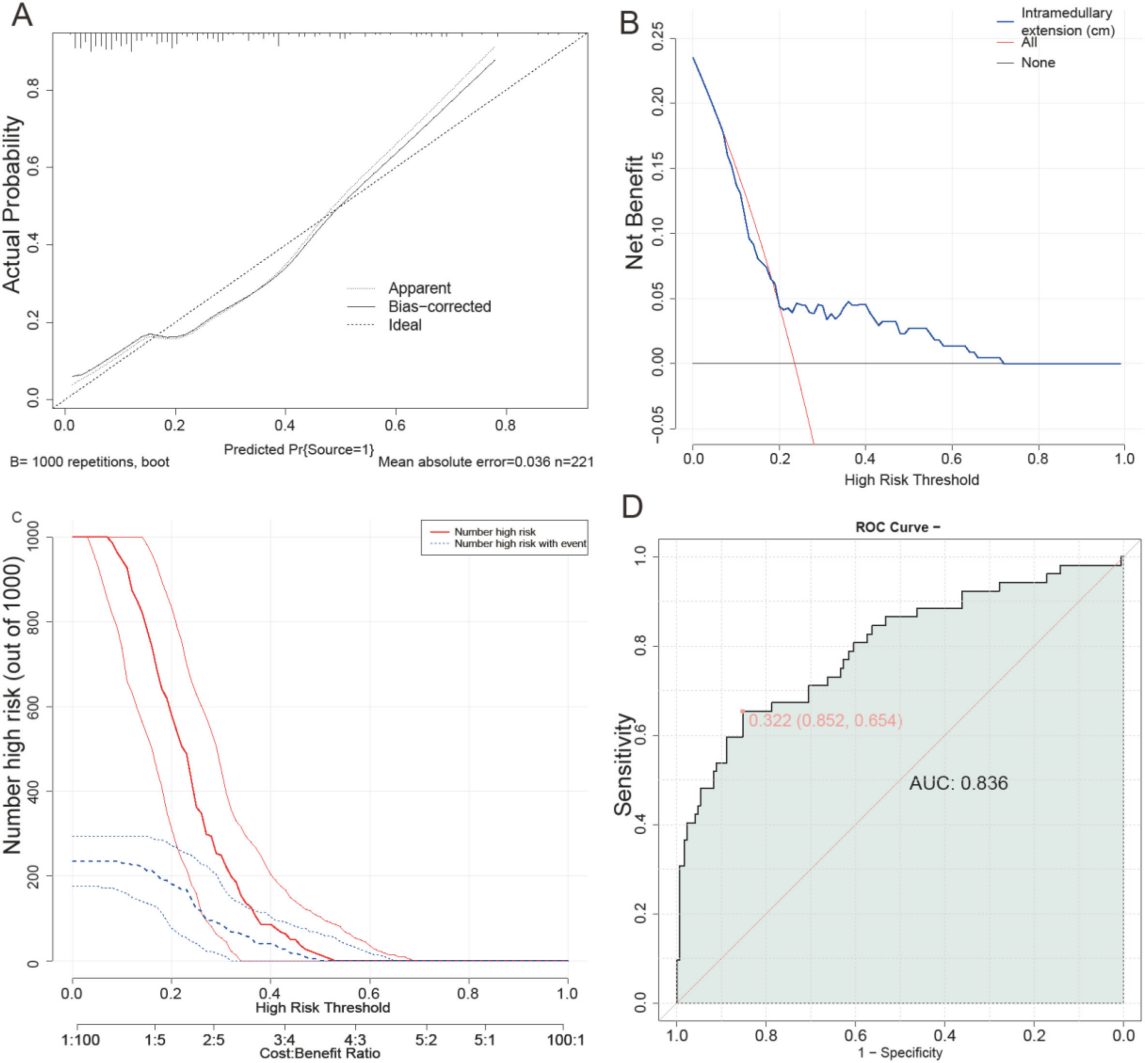
Development of a prediction model for multimodal MRI quantitative features influencing poor prognosis of non-metastatic primary osteosarcoma. **(A)** calibration curve; **(B)** decision curve; **(C)** clinical impact curve; **(D)** ROC curve. AUC, Area Under the Curve; ROC, Receiver Operating Characteristic.

**Figure 5** illustrates the development of a prediction model for multimodal MRI quantitative features influencing poor prognosis in non-metastatic primary osteosarcoma. Panel A presents a calibration curve demonstrating the agreement between predicted and actual outcomes across various probability thresholds. Panel B shows a decision curve analysis, highlighting the net benefit of using the model at different threshold probabilities to guide clinical decisions. Panel C features a clinical impact curve, depicting how varying the high-risk threshold affects the number of individuals classified as high risk versus those who truly experienced the event out of 1000 cases. Lastly, Panel D displays an ROC curve with an AUC of 0.836, illustrating the model’s ability to discriminate between patients with different prognoses effectively. Together, these panels provide a comprehensive evaluation of the model’s performance in terms of calibration, clinical utility, impact on risk stratification, and discrimination power. The calibration curve demonstrated good agreement between predicted and observed outcomes. The Hosmer-Lemeshow test yielded a P value of 0.391, indicating no significant lack of fit. The Brier score was 0.162, further supporting adequate model calibration.

**Figure 6:**
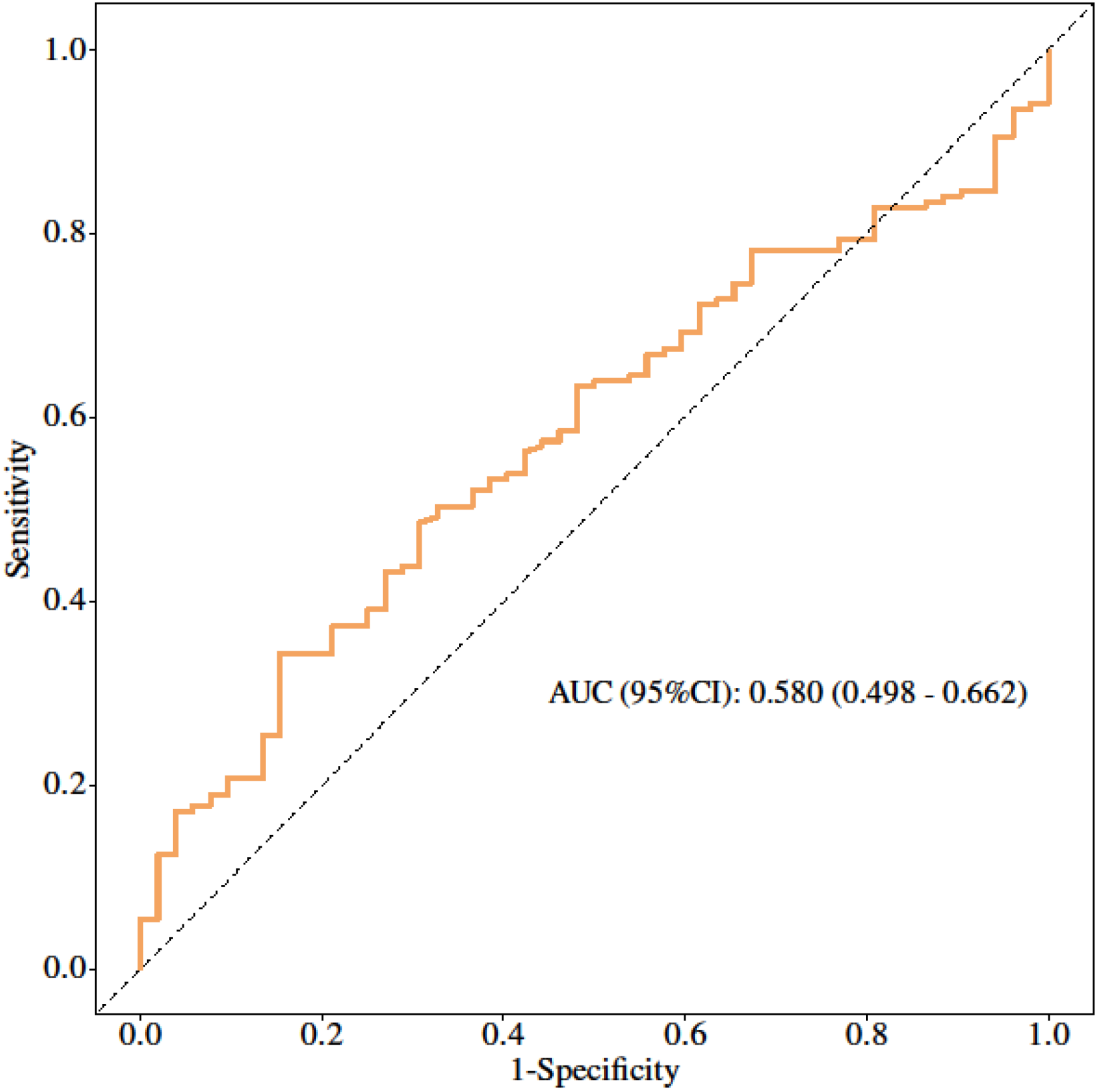
ROC curves of the clinical baseline model.

To evaluate the added predictive value beyond conventional clinical factors, a clinical baseline model incorporating Enneking stage and tumor volume was constructed (**Figure 6**). This baseline model achieved an AUC of 0.580, which was lower than that of the multimodal MRI model, demonstrating the superior discriminative ability of the proposed imaging model.

### 3.7 External validation of the predictive model

In the external validation set comparing general information between the Good Prognosis Group and Poor Prognosis Group, several P values indicated no significant differences across most parameters (**Table 7**). Age showed no significant difference (P = 0.836), nor did BMI (P = 0.457), gender (P = 0.312), education level (P = 0.275), family history (P = 0.749), residence (P = 0.530), Han ethnicity (P = 0.836), smoking (P = 0.188), drinking (P = 0.299), site of tumor (P = 0.820), Enneking stage (P = 0.612), pathological fracture (P = 0.145), pathology (P = 0.698), course of disease duration (P = 0.864), or follow-up period (P = 0.665). These findings indicate that the baseline characteristics and clinical features are similarly distributed between patients with good and poor prognoses in this external test set, supporting the applicability and reliability of the predictive model across different populations.

**Table 7:** Comparison of general information between two groups in the external test set.

Parameters	Good prognosis group (n=65)	Poor prognosis group (n=27)	t/χ^2^	P
Age [n (%)]			0.043	0.836
- <18 years old	37 (56.92%)	16 (59.26%)		
- ≥18 years old	28 (43.08%)	11 (40.74%)		
BMI (kg/m^2^)	22.58 ± 3.14	23.14 ± 3.52	0.748	0.457
Gender [n (%)]			1.021	0.312
- Male	34 (52.31%)	11 (40.74%)		
- Female	31 (47.69%)	16 (59.26%)		
Education level [n (%)]			1.193	0.275
- Secondary School or below	37 (56.92%)	12 (44.44%)		
- College or above	28 (43.08%)	15 (55.56%)		
Family History [n (%)]	8 (12.31%)	2 (7.41%)	0.102	0.749
Residence [n (%)]			0.394	0.530
- Urban	43 (66.15%)	16 (59.26%)		
- Rural	22 (33.85%)	11 (40.74%)		
Han ethnicity [n (%)]	55 (84.62%)	24 (88.89%)	0.043	0.836
Smoking [n (%)]	15 (23.08%)	3 (11.11%)	1.736	0.188
Drinking [n (%)]	16 (24.62%)	4 (14.81%)	1.077	0.299
Site of tumor [n (%)]			0.245	0.820
- Femur	35 (53.85%)	16 (59.26%)		
- Tibia/fibula	11 (16.92%)	7 (25.93%)		
- Humerus	9 (13.85%)	2 (7.41%)		
- Pelvis	6 (9.23%)	1 (3.70%)		
- Head/neck	3 (4.62%)	1 (3.70%)		
- Other	1 (1.54%)	0 (0.00%)		
Enneking stage [n (%)]			1.813	0.612
- IA	6 (9.23%)	5 (18.52%)		
- IB	13 (20.00%)	6 (22.22%)		
- IIA	25 (38.46%)	9 (33.33%)		
- IIB	21 (32.31%)	7 (25.93%)		
Pathological fracture [n (%)]	16 (24.62%)	3 (11.11%)	2.123	0.145
Pathology [n (%)]			0.151	0.698
-Osteoblastic	11 (16.92%)	3 (11.11%)		
-Others	54 (83.08%)	24 (88.89%)		
Course of Disease (months)	4.53 ± 1.42	4.58 ± 1.31	0.172	0.864
Follow-up period (months)	18.31 ± 1.01	18.42 ± 1.31	0.435	0.665

BMI, Body Mass Index.

In the comparison of differential factors between the Good Prognosis Group and Poor Prognosis Group in the external test set, several significant differences were noted (**Table 8**). The contrast agent back-flux rate constant (Kep) was significantly higher in the Good Prognosis Group (P = 0.004). Intramedullary extension was significantly greater in the Poor Prognosis Group (P = 0.021). The pure diffusion coefficient (D value) was significantly higher in the Good Prognosis Group (P = 0.036). The pseudo-diffusion coefficient (D* value) was also significantly higher in the Good Prognosis Group (P = 0.017). Lastly, the Apparent Diffusion Coefficient (ADC value) was significantly higher in the Good Prognosis Group (P = 0.015). These findings indicate that patients with a good prognosis had significantly higher levels of Kep, D value, D* value, and ADC value compared to those with poor prognosis. Conversely, the Poor Prognosis Group showed a significantly greater intramedullary extension.

**Table 8:** Comparison of differential factors between two groups in the external test set.

Parameters	Good prognosis group (n=65)	Poor prognosis group (n=27)	t	P
K_ep_ (min^-1^)	1.35 ± 0.13	1.26 ± 0.15	2.932	0.004
Intramedullary extension (cm)	9.16 ± 1.53	10.32 ± 2.31	2.408	0.021
D value (×10–^3^ mm^2^/s)	0.97 ± 0.42	0.81 ± 0.27	2.141	0.036
D* value (×10–^3^ mm^2^/s)	19.35 ± 5.31	16.53 ± 4.52	2.422	0.017
ADC value (×10–^3^ mm^2^/s)	1.21 ± 0.41	1.04 ± 0.24	2.483	0.015

K_ep_, contrast agent back-flux rate constant; D value, pure diffusion coefficient; D* value, pseudo-diffusion coefficient; ADC, Apparent Diffusion Coefficient.

### 3.10 External validation ROC

**Figure 7:**
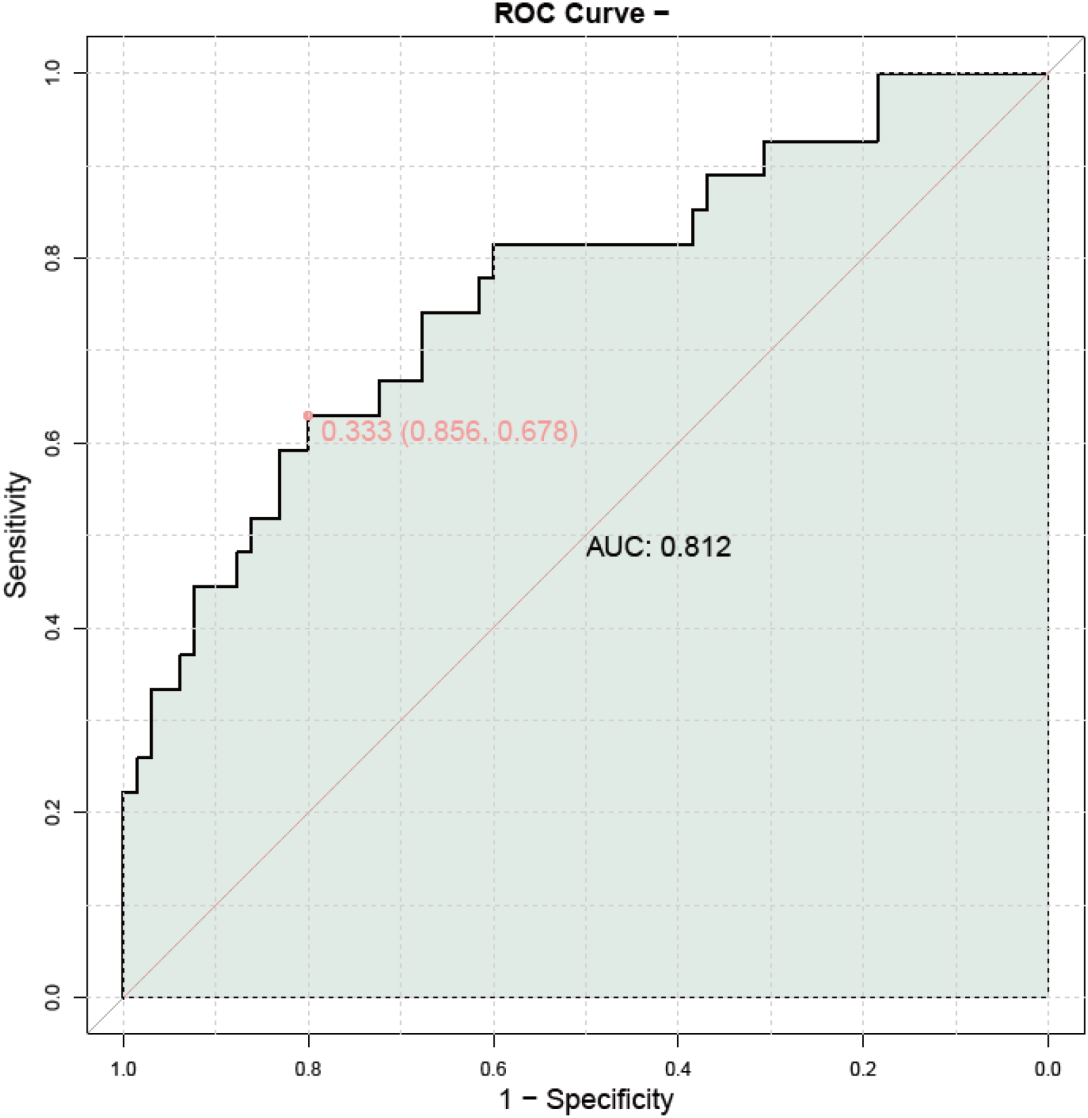
External validation ROC curve. AUC, Area Under the Curve; ROC, Receiver Operating Characteristic.

In the external validation ROC curve depicted, the model’s discriminatory ability is illustrated with an Area Under the Curve (AUC) of 0.812, indicating a good level of discrimination between the Good Prognosis Group and Poor Prognosis Group (**Figure 7**). The curve plots sensitivity against 1-specificity, showing the trade-off between true positive rate and false positive rate at various threshold settings. A point on the curve at a 1-specificity of approximately 0.333 corresponds to a sensitivity of around 0.678, suggesting that at this particular threshold, the model achieves a balance between correctly identifying patients with poor prognosis while minimizing false positives. The proximity of the ROC curve to the top-left corner of the plot further supports the model’s effectiveness in distinguishing between the two groups. This external validation confirms the robustness and generalizability of the predictive model across different datasets.

## 4 Discussion

In this study, we successfully developed and externally validated a multimodal MRI predictive model for prognosis of non-metastatic primary osteosarcoma. By integrating quantitative features from T1-weighted dynamic contrast-enhanced (DCE), T2-weighted anatomical, and diffusion-weighted imaging (DWI) sequences, the model effectively distinguished between patients with good and poor prognosis. Importantly, this non-invasive approach provided a tool to identify high-risk patients before treatment began, addressing the current clinical practice’s reliance on postoperative histopathological assessment for prognosis. Validation across independent cohorts highlighted the model’s potential universality and clinical applicability.

Our study results showed that MRI could capture specific aspects of tumor pathophysiology in assessing osteosarcoma aggressiveness. Lower pure diffusion coefficient (D value), pseudo-diffusion coefficient (D* value), and apparent diffusion coefficient (ADC value) obtained through DWI demonstrated a strong correlation with poor prognosis. This provided robust evidence for the role of high cell density and restricted water diffusion in aggressive tumor biology (22, 23). Typically, lower ADC and D values correlated with dense cellularity and reduced extracellular space, characteristics of rapidly proliferating high-grade malignancies (24, 25). Hypothetically, the high cell density and restricted diffusion reflected by low ADC values may be associated with enhanced tumor cell proliferation and survival mechanisms, which could contribute to treatment resistance and a higher likelihood of metastasis (26). Similarly, lower D values indicated impaired pseudo-diffusion related to microcirculatory perfusion, suggesting issues with capillary blood flow within the osteosarcoma tumor microenvironment (27). This might indicate chaotic and inefficient neoangiogenesis or increased interstitial pressure hindering perfusion, both associated with treatment resistance and poor outcomes. Our findings supported previous research indicating that DWI parameters, particularly ADC, served as biomarkers for osteosarcoma grading and treatment response (28). However, by incorporating IVIM-derived parameters (D and D*) into the prognostic model for non-metastatic disease and examining their independent significance alongside ADC, we added new perspectives. This approach offered additional insights into different microenvironmental factors contributing to poor outcomes, such as cellularity and perfusion.

In DCE-MRI, the importance of the contrast agent reverse flux rate constant (Kep) provided another key dimension. Our model found that lower Kep values indicated poor prognosis. Kep mainly reflected the rate at which contrast agent returned from the extravascular extracellular space (EES) to the vascular system (29). When Kep values were lower, it meant that the clearance of contrast agent in the tumor stroma was slower (30). Hypothetically, this situation could be related to several pathophysiological mechanisms in aggressive tumors. Aggressive tumors often had more tortuous and less efficient vascular systems (31). These poorly formed, leaky vessels might hinder effective contrast agent clearance. High cell density, abundant extracellular matrix (ECM), and dysfunctional lymphatics increased interstitial fluid pressure (IFP), further impeding the return of contrast agent to capillaries (32). These series of changes could collectively contribute to the formation of a pro-fibrotic and immunosuppressive microenvironment, which may in turn facilitate tumor progression (33). Additionally, although Kep primarily related to the vasculature and EES, changes in cellular metabolism or membrane integrity could also indirectly affect interstitial dynamics (34). We observed that lower Kep values correlated with poor prognosis, consistent with findings from other studies on sarcomas and other solid tumors (35). Research indicated that in highly aggressive tumors, abnormal and dysfunctional microvascular features such as slow blood flow and impaired clearance, rather than simple high permeability, were key factors in treatment resistance. This highlighted the complexity and context-specific importance of DCE-MRI parameters and underscored the need to develop disease-specific models.

Additionally, the measurement of intramedullary extension using T2-weighted imaging emerged as a strong and independent predictor of poor prognosis. This anatomical feature directly reflected the longitudinal spread of the tumor within the bone marrow cavity. Greater intramedullary extension typically indicated more extensive bone marrow infiltration, a known marker of poor prognosis in surgical and pathological studies (36). When the extent of bone marrow involvement was larger, achieving wide surgical margins became more challenging, increasing the risk of skip metastasis and suggesting that the tumor had stronger local invasiveness and colonization ability (37). Hypothetically, extensive bone marrow invasion may be associated with tumor cells acquiring enhanced motility, invasiveness, and colonization abilities, which could promote tumor cell dissemination and survival within the bone marrow niche (38). As demonstrated in our study, MRI could accurately quantify this feature before treatment, providing a crucial non-invasive method to assess local tumor burden and invasiveness. This approach complemented the functional information provided by DWI and DCE-MRI and bridged the gap between traditional anatomical staging and advanced sequence-based functional/biological assessments.

The integration of these different but complementary MRI biomarkers, including those reflecting cell density (DWI), microvascular function and permeability (DCE-MRI), and local invasiveness (T2 anatomical imaging), formed the core innovation and advantage of our model. While individual parameters showed only moderate discriminative ability in ROC analysis, their combination within a multivariate logistic regression framework significantly improved the model’s overall performance. This synergistic effect suggested that poor prognosis in osteosarcoma was driven by multiple biological processes, such as high cell proliferation, perfusion dysfunction, impaired interstitial clearance, and aggressive local spread (39). Compared to relying on single parameters or postoperative histological assessments, capturing these multifaceted pathophysiological features non-invasively before treatment offered substantial advantages. These key features consistently distinguished prognostic groups in both the training and external validation sets, further enhancing their biological and clinical relevance. Our model not only performed well in calibration but also demonstrated positive net benefits across a range of clinically relevant risk thresholds in decision curve analysis, indicating good agreement between predicted and actual risks.

Despite the encouraging results, this study had several limitations that warrant attention. First, the single-center design introduced potential selection bias and limited the model’s generalizability across different patient populations and imaging protocols. Although external validation partly addressed this issue, broader multicenter prospective validation was essential. Additionally, the retrospective nature of the study introduced biases that could affect the validity of the conclusions. Variations in MRI scanners and acquisition protocols, even within a single center, could impact the stability of radiomic features. While efforts were made to standardize post-processing, future studies should more rigorously coordinate protocols or use calibration methods to address these issues. Internal cross-validation or bootstrapping was not performed. Given the small sample size of the poor-prognosis subgroup, there is a risk of model overfitting. However, external validation provides a degree of reliability. In this study, a 30-month period was used as the primary endpoint for prognosis assessment. Although this time point captures the critical clinical window during which most recurrence events in osteosarcoma occur, and radiological responses defined by RECIST have been confirmed to be closely related to long-term survival, it may not be sufficient to comprehensively assess the long-term outcomes of osteosarcoma. The European Society for Medical Oncology (ESMO) guidelines recommend a total follow-up period of 5 to 10 years or more for high-grade osteosarcoma to monitor late recurrences and treatment-related late toxicities (40). Therefore, a 30-month follow-up may fail to identify late recurrence events, potentially overestimating the positive predictive value of the model and underestimating the true risk for some high-risk patients. In the future, it will be necessary to extend the follow-up time and use overall survival and event-free survival as endpoints to further validate the prognostic value of the model. Our model currently considered only clinical and imaging variables. Integrating known molecular and genetic biomarkers that influence osteosarcoma prognosis could further enhance predictive accuracy and provide deeper biological insights, leading to a true multi-omics prognostic tool. Finally, our definition of “poor prognosis” primarily relied on recurrence or metastasis during the follow-up period, which, while clinically relevant, could be refined. Integrating pathological necrosis rates after neoadjuvant chemotherapy and endpoints such as event-free survival can more finely and biologically meaningfully define ‘adverse prognosis’ from both histological response and long-term clinical outcomes. This integration could potentially enhance the model’s discriminative ability and clinical relevance.

Future research directions should prioritize addressing these limitations. Advancing the integration of multi-omics is key to deepening the value of the model. Future work could explore combining the radiomic features from this study with molecular genetic markers of tumors to build a more comprehensive predictive model. This integration faces dual challenges of methodology and data. Methodologically, advanced algorithms capable of integrating high-dimensional heterogeneous data need to be developed. On the data level, systematic solutions are required to address issues such as bio-sample acquisition across multiple centers, standardization of molecular testing, and consistency in multi-modal data registration and annotation. Standardized imaging protocols across different platforms and multicenter prospective validation are crucial for confirming the robustness of the model and promoting its clinical adoption. To achieve reproducible radiomic analysis, future studies should clearly report scanning parameters, reconstruction algorithms, and phantom calibration details in accordance with international standards such as the Image Biomarker Standardisation Initiative (IBSI). Additionally, exploring the use of deep learning techniques like generative adversarial networks (GANs) for cross-center data harmonization could be beneficial. Standardized imaging protocols across different platforms and multicenter prospective validation were crucial for confirming model robustness and promoting clinical adoption. Investigating the temporal changes of these radiomic features during neoadjuvant chemotherapy was another important direction. This could help identify biomarkers for early treatment response assessment and allow for adaptive treatment modifications based on early patient responses, leading to more personalized treatment strategies. Developing fully automated segmentation and feature extraction methods using deep learning was also essential. Such methods could enhance reproducibility and efficiency, and potentially uncover novel and complex imaging features beyond traditional radiological characteristics. Finally, clinical utility studies were needed to evaluate how this predictive model impacted actual patient management decisions. A feasible approach is to conduct pragmatic clinical trials or prospective cohort studies, grouping patients based on the model’s prediction of high-risk and low-risk categories, and evaluating whether they follow differentiated treatment pathways based on model risk stratification. The primary endpoints should include rates of changes in physician decision-making, treatment adherence, and key long-term patient outcomes such as event-free survival and overall survival. By comparing the differences in these endpoints between the model-guided group and the standard treatment group, the clinical net benefit of the tool can be empirically assessed.

## 5 Conclusion

In summary, this study successfully developed and validated a robust multimodal MRI radiological model for predicting pre-treatment prognosis in non-metastatic primary osteosarcoma. By integrating critical pathophysiological features (including high cellularity, dysfunctional perfusion, impaired interstitial clearance, and extensive bone marrow infiltration) the model serves as a powerful non-invasive tool for identifying high-risk patients. This early identification allows for timely intervention, enabling intensified treatment strategies or participation in innovative therapeutic trials aimed at addressing the aggressive nature of the disease. The results highlight the potential of this model to enhance clinical decision-making and improve patient outcomes in osteosarcoma management.

## Data Availability

The raw data supporting the conclusions of this article will be made available by the authors, without undue reservation.
